# *Cryptococcus neoformans*, a global threat to human health

**DOI:** 10.1186/s40249-023-01073-4

**Published:** 2023-03-17

**Authors:** Youbao Zhao, Leixin Ye, Fujie Zhao, Lanyue Zhang, Zhenguo Lu, Tianxin Chu, Siyu Wang, Zhanxiang Liu, Yukai Sun, Min Chen, Guojian Liao, Chen Ding, Yingchun Xu, Wanqing Liao, Linqi Wang

**Affiliations:** 1 College of Veterinary Medicine, Henan Agricultural University, Zhengzhou, 450046 Henan China; 2 State Key Laboratory of Mycology, Institute of Microbiology, Chinese Academy of Sciences, Beijing, 100101 China; 3 University of Chinese Academy of Sciences, Beijing, 100039 China; 4 Department of Dermatology, Shanghai Key Laboratory of Molecular Medical Mycology, Changzheng Hospital, Shanghai, 200003 China; 5 The Medical Research Institute, College of Pharmaceutical Sciences, Southwest University, Chongqing, 400715 China; 6 College of Life and Health Sciences, Northeastern University, Shenyang, 110819 Liaoning China; 7 Department of Laboratory Medicine, and Beijing Key Laboratory for Mechanisms Research and Precision Diagnosis of Invasive Fungal Diseases, Peking Union Medical College Hospital, Chinese Academy of Medical Sciences, Beijing, 100730 China

**Keywords:** Fungal infections, *Cryptococcus neoformans*, Cryptococcal meningitis, Antifungal resistance, Antifungal tolerance

## Abstract

**Background:**

Emerging fungal pathogens pose important threats to global public health. The World Health Organization has responded to the rising threat of traditionally neglected fungal infections by developing a Fungal Priority Pathogens List (FPPL). Taking the highest-ranked fungal pathogen in the FPPL, *Cryptococcus neoformans*, as a paradigm, we review progress made over the past two decades on its global burden, its clinical manifestation and management of cryptococcal infection, and its antifungal resistance. The purpose of this review is to drive research efforts to improve future diagnoses, therapies, and interventions associated with fungal infections.

**Methods:**

We first reviewed trends in the global burden of HIV-associated cryptococcal infection, mainly based on a series of systematic studies. We next conducted scoping reviews in accordance with the guidelines described in the Preferred Reporting Items for Systematic Reviews and Meta-analyses extension for Scoping Reviews using PubMed and ScienceDirect with the keyword *Cryptococcus neoformans* to identify case reports of cryptococcal infections published since 2000. We then reviewed recent updates on the diagnosis and antifungal treatment of cryptococcal infections. Finally, we summarized knowledge regarding the resistance and tolerance of *C. neoformans* to approved antifungal drugs.

**Results:**

There has been a general reduction in the estimated global burden of HIV-associated cryptococcal meningitis since 2009, probably due to improvements in highly active antiretroviral therapies. However, cryptococcal meningitis still accounts for 19% of AIDS-related deaths annually. The incidences of CM in Europe and North America and the Latin America region have increased by approximately two-fold since 2009, while other regions showed either reduced or stable numbers of cases. Unfortunately, diagnostic and treatment options for cryptococcal infections are limited, and emerging antifungal resistance exacerbates the public health burden.

**Conclusion:**

The rising threat of *C. neoformans* is compounded by accumulating evidence for its ability to infect immunocompetent individuals and the emergence of antifungal-resistant variants. Emphasis should be placed on further understanding the mechanisms of pathogenicity and of antifungal resistance and tolerance. The development of novel management strategies through the identification of new drug targets and the discovery and optimization of new and existing diagnostics and therapeutics are key to reducing the health burden.

## Background

Emerging fungal pathogens and infections pose increasing threats to global public health. People most at risk of invasive fungal disease (IFD) are those with a compromised immune system, due to HIV infection, chemotherapy and immunotherapy for cancer, solid organ transplantation, or other factors. In addition, people with underlying diseases including diabetes mellitus, liver or kidney disease, chronic obstructive pulmonary disease, and viral respiratory tract infections, have been newly identified as an at-risk population.

Viral and bacterial infections tend to be the pathogens that receive the most attention, especially for their potential to cause pandemics; accordingly, human fungal pathogens and IFDs have long been underrecognized. In recent years, however, this perception has changed rapidly, and there is growing concern about the threats posed by fungi to animals and humans. For example, pathogenic species belonging to the phylum Chytridiomycota have been shown to be a major factor leading to the extinction of multiple amphibian species [1, 2]. In addition to the emergence of amphibian fungal pathogens, adaptation to increased temperatures due to climate change has led to the ability of multiple fungal species to overcome mammalian endothermic defenses, leading to their establishment as new human fungal pathogens [3, 4]. Climate change has also altered or expanded the geographic distribution of known pathogenic fungal species, resulting in the emergence of diseases in regions where they were not been previously reported [5]. Officials have already recognized and responded to the rapid increase in the emergence of new fungal pathogens. For instance, an updated Catalogue of Microbial Pathogens Transmitted to Humans released by the National Health Commission of China in 2022 included 107 new fungal pathogens, whereas only 12 new viruses and 4 new species of bacteria were added [6]. Intriguingly, certain fungal pathogens have recently been associated with coronavirus disease 2019 (COVID-19) [7–10] and cancer developments [11, 12].

The mortality rates of infections caused by known or new pathogenic fungi are often high. Underdiagnosis and the extreme lack of treatment options are important reasons for the high mortality rates [13]. Approved treatments for human fungal infections are limited to four classes of antifungal agents: polyenes, flucytosine, echinocandins, and azoles. Unfortunately, the wide-spread use of existing antifungals in both medicine and agriculture has greatly accelerated the acquisition and emergence of antifungal resistance, which is fundamentally an evolutionary response to selective pressures. The development of new antifungal agents is hampered by the similarity between fungal cells and the cells of their mammalian host, since molecules toxic to fungi tend to be toxic to humans. Because the prevalence of fungal infections has increased and has emerged as a pressing threat to public health, antifungal resistance has now been officially recognized by the listing of fungal pathogens on the Antibiotic Resistance Threats Report produced by the Centers for Disease Control and Prevention (USA) in 2019 [14]. The emergence of drug-resistant strains has further increased the threat of fungal infections; resistance to all four types of antifungal agents has been documented in clinical isolates of fungal pathogens. In response to the rising threats of fungal infections and antifungal resistance, the World Health Organization (WHO) released the Fungal Priority Pathogens List (FPPL) in October 2022 to focus and drive further research, surveillance, and policy interventions [15]. In the WHO FPPL, 19 fungal pathogens were ranked and categorized as critical, high, or medium priority pathogens (Table 1). In particular, *Cryptococcus neoformans*, *Candida auris*, *Aspergillus fumigatus*, and *Candida albicans* were ranked as “critical” fungal pathogens based on their antifungal resistance, mortality rates, lack of evidence-based diagnostic and treatment options, annual incidence, and complications and sequelae.

**Table 1:** WHO fungal priority pathogens list

Pathogen	Final ranking of pathogens	Geographic distribution	Mortality
Critical priority group			
* Cryptococcus neoformans*	1	Global	41–61%
* Candida auris*	2	Global	29–53%
* Aspergillus fumigatus*	3	Global	47–88%
* Candida albicans*	4	Global	20–50%
High priority group			
* Nakaseomyces glabrata* (*Candida glabrata*)	5	Global	20–50%
* Histoplasma* spp.	6	Global	21–53% (HIV/AIDS patients) 9–11% (immunosuppressed patients)
* Eumycetoma causative agents*	7	Global	Lack of data. Thought to be low
* Mucorales*	8	Global	23–80% (adult patients) 72.7% (pediatric patients)
* Fusarium* spp.	9	Global	43–67%
* Candida tropicalis*	10	Global	55–60% (adult patients) 26–40% (pediatric patients)
* Candida parapsilosis*	11	Global	20–45%
Medium priority group			
* Scedosporium* spp.	12	Global	42–46%
* Lomentospora prolificans*	13	Global	50–71% (adult patients) 50% (immunocompromised children)
* Coccidioides* spp.	14	Americas	2–13%
* Pichia kudriavzeveii* (*Candida krusei*)	15	Global	44–67%
* Cryptococcus gattii*	16	Global	10–23% (CNS infections) 15–21% (pulmonary infections)
* Talaromyces marneffei*	17	South-East Asia, China	12–21%
* Pneumocystis jirovecii*	18	Global	0–100%
* Paracoccidioides* spp.	19	Central and South America	3–23%

*Source* WHO fungal priority pathogens list to guide research, development and public health action. *CNS* Central nervous system

*Cryptococcus neoformans*, the top-ranked fungal pathogen in the WHO FPPL, is a globally distributed opportunistic fungal pathogen that is primarily of environmental origin and that can cause life-threatening cryptococcosis. *C. neoformans* contain two varieties: *C. neoformans* var. neoformans and *C. neoformans* var. grubii. A third variety, *C. neoformans* var. gattii, was later defined as a distinct species, *Cryptococcus gattii*. The most recent classification system divides these varieties into seven species [16, 17]. *C. neoformans* refers to *C. neoformans* var. grubii. A new species name, *Cryptococcus deneoformans*, is used for the former *C. neoformans* var. neoformans. *C. gattii* is divided into five species. Hence, for the ease of description and discussion, we use *C. neoformans* to refer to both *C. deneoformans* and *C. neoformans*. The mortality rate of cryptococcosis is alarmingly high, especially in patients with HIV infection, in whom the mortality rate ranges from 41% to 61%. Similar to most other fungal infections, access to diagnosis and treatment of cryptococcosis is limited in many countries with developing healthcare systems. Another important issue is that antifungal-resistant clinical isolates have been emerging rapidly, and the mechanisms of antifungal resistance are far from being fully understood. Taking *C. neoformans* as a paradigm, in this study we review the progress made over the past two decades on the global burden, clinical manifestation and management of cryptococcal infection and on antifungal resistance and tolerance. The purpose of this review is to highlight the emerging threats posed by fungal infections and to drive research efforts in fungal infections to improve future diagnoses, therapies, and interventions.

## Methods

### Literature search strategy

This scoping review was conducted in accordance with the guidelines described in the Preferred Reporting Items for Systematic Reviews and Meta-analyses extension for Scoping Reviews (PRISMA-ScR) [18]. To identify and select cases and studies for inclusion, a systematic literature search was performed in PubMed and ScienceDirect using the keyword *Cryptococcus neoformans* for studies published in English since 2000. We used Endnote 20 (Clarivate, Philadelphia, USA) to manage the articles.

### Study eligibility criteria and data extraction

Conference abstracts and editorials were excluded, and case reports and research articles were included for review. To be included, the case reports must meet the following criteria: (1) be identified as case reports; (2) include sufficient details about patients; (3) refer to disease states in which *C. neoformans* was the causative pathogen; (4) provide sufficient details about clinical manifestations; (5) describe treatment details including the specific antifungal therapy administered, if applicable; and (6) describe patient outcomes. The full text of articles that met the inclusion criteria was then searched. Exclusion criteria included studies involving patients under 18 years of age, duplicate studies, and studies for which the full text was not accessible.

Screening of the results of the literature search against the eligibility criteria for case reports and research articles was performed by two independent authors, and any disagreement was mediated by a third author. Failure to reach a consensus was resolved by senior authors. Two groups of authors (group 1: FZ, ZL, and TC; group 2: SW, YS, and ZL) independently extracted information from eligible case reports. The extracted information included first author, year of publication, patient information (country, age, sex, medical or surgical history), clinical manifestation and duration of the infection, site of infection, course of treatment and patient outcome.

## Results

### Trends in the global burden of HIV-associated cryptococcal infection

*Cryptococcus neoformans* is a globally distributed opportunistic fungal pathogen of primarily environmental origin, commonly associated with bird feces (especially pigeon feces), soil, and decaying wood. Humans and animals typically acquire the infection from inhaling dust contaminated with bird feces, but direct transmission of cryptococcosis between humans or between animals has not been reported. After inhalation of fungal basidiospores or desiccated yeast cells from the environment, cryptococcal infection initially occurs in the lungs (cryptococcal pneumonia), followed by dissemination of fungal cells to the central nervous system (CNS) (cryptococcal meningitis, CM) and blood (cryptococcemia), which may be achieved through a Trojan horse mechanism. The majority of patients are immunocompromised, with the most significant risk factor being HIV infection.

A series of landmark studies were published in 2009 [19], 2017 [20], and 2022 [21]. Those systematic studies respectively estimated the global and regional burden of HIV-associated CM in 2007, 2014, and 2020, based on available incidence data in HIV-infected cohorts (mainly from the Joint United Nations Programme on HIV/AIDS) and population-based HIV impact assessment surveys. Notably, with the development of improved antiretroviral treatments and systemic antifungal treatments, the global incidence of HIV-associated CM declined from 960,000 cases in 2007 to 220,000 in 2014, and further to 150,000 in 2020 (Table 2). Despite the downward trend in the burden of cryptococcosis, the incidence of cryptococcal infections in immunocompromised individuals remains high (Table 2). For instance, in studies published in 2017 and 2022, the prevalence of cryptococcal antigenemia was estimated to be 6.0% among people with CD4 cell counts of less than 100 cells/µl and 4.4% among HIV-positive people with CD4 cell counts of less than 200 cells/µl, corresponding to 280,000 and 180,000 cases of cryptococcal antigenemia (Table 2), respectively.

**Table 2:** Estimates of the global prevalence of cryptococcal meningitis and associated death

	CrAg positive cases (in thousands)^a^		CM cases (in thousands)			CM deaths (in thousands)		
	2014	2020	2007	2014	2020	2007	2014	2020
Global	278.0 (195.5–341.0)^b^	179.0 (133.0–219.0)	957.9 (371.7–1544)	223.1 (150.6–282.4)	152.0 (111.0–185.0)	624.7 (125.0–1124.9)	181.1 (119.4–234.3)	112.0 (79.0–134.0)
Sub-Saharan Africa	204.3 (148.4–237.8)	97.0 (73.0–120.0)	720.0 (144.0–1300.0)	162.5 (113.6–193.9)	82.0 (61.0–101.0)	504.0 (100.8–907.2)	135.9 (93.9–163.9)	71.0 (52.0–88.0)
Asia and the Pacific	52.3 (32.9–74.1)	51.0 (42.0–60.0)	133.6 (26.7–240.5)	43.2 (25.3–64.7)	44.0 (35.0–51.0)	67.2 (13.4–121)	39.7 (20.6–59.7)	26.0 (21.0–30.0)
Latin America	7.0 (3.6–11.1)	14.0 (10.0–17.0)	54.4 (10.9–97.9)	5.3 (2.6–8.9)	12.0 (9.0–14.0)	29.9 (6.0–53.8)	2.4 (1.1–4.4)	7.0 (5.0–9.0)
Europe^c^ and North America	8.9 (7.0–11.1)	15.0 (13.0–17.0)	34.5 (7.1–64.0)	7.4 (5.7–9.3)	12.0 (10.5–1.4)	15.8 (3.1–28.4)	2.5 (1.8–3.4)	7.0 (5.7–8.4)
Caribbean	1.8 (1.3–2.2)	2.0 (1.7–2.3)	7.8 (1.6–14.1)	1.4 (1.0–1.8)	1.7 (1.4–1.9)	4.3 (0.9–7.8)	0.7 (0.5–0.9)	1.0 (0.8–1.0)
Middle East and North Africa	3.6 (2.6–5.0)	0.5 (0.1–0.6)	6.5 (11.3–7.6)	3.3 (2.4–4.5)	0.4 (0.1–0.5)	3.6 (0.7–6.4)	1.9 (1.3–2.7)	0.2 (0.1–0.3)

*CrAg* Cryptococcal antigen; *CM* Cryptococcal meningitis

^a^Antigenemia data was not available for estimation of cases in 2007

^b^Data provided as mean (95% confidence interval)

^c^The 2020 data contains cases from Central Asia

Consistent with global trends, the annual incidence of CM in sub-Saharan Africa has been declining (720,000 in 2007, 162,500 in 2014, and 82,000 in 2020), although this region has had, and continues to have, the greatest burden of cryptococcal infection (Table 2). The region with the second highest number of cases is Asia and the Pacific, where the incidence has seemingly reached a steady state (43,200 in 2014 and 44,000 in 2020) with a reduction in CM-associated deaths (from 39,700 to 2014 to 26,000 in 2020) (Table 2). Of note, the incidence of CM has increased by approximately twofold in Europe and North America (from 7400 to 2014 to 12,000 in 2020) and in Latin America (from 5300 to 2014 to 12,000 in 2020). These data highlight the need for further evaluation and refinement of the current control and prevention protocols in these two regions, even though the total burdens are currently at relatively low levels.

In general, there has been a significant geographic redistribution of the estimated global burden of HIV-associated CM since 2009, probably due to the improvement and expansion of highly active antiretroviral therapies (HAART). However, CM still accounts for 19% of AIDS-related deaths annually, according to estimates for both 2017 [20] and 2020 [21]. Although these series of studies have led to a systematic understanding of HIV-associated cryptococcal infections, it is still not possible to accurately assess the total annual incidence and mortality, given the wide variations in the diagnoses and treatments in different regions of the world.

### Clinical manifestations of cryptococcal infection

**Fig. 1:**
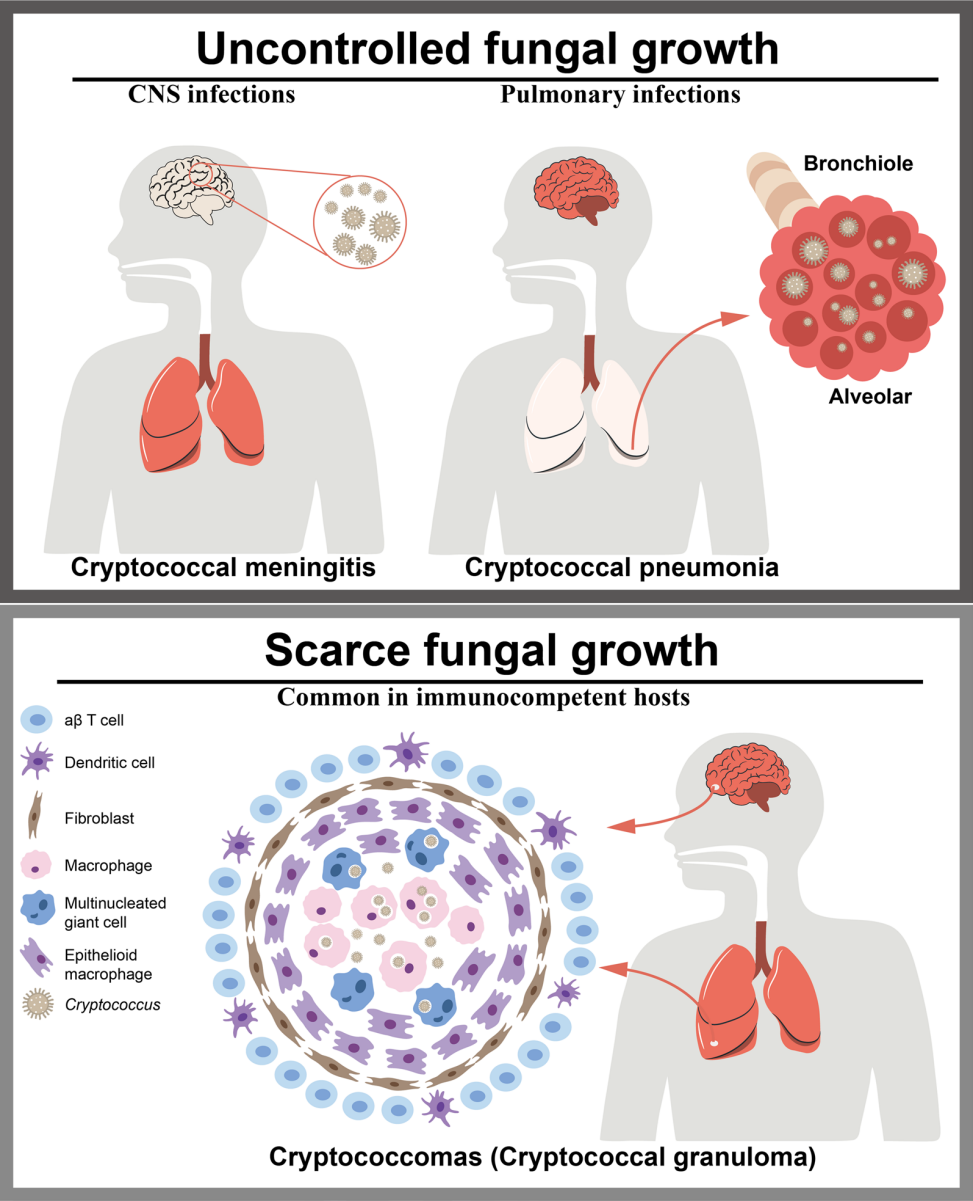
Clinical manifestation of cryptococcal infection. The most common clinical manifestation of cryptococcal infection are CNS infections, which cause cryptococcal meningitis (Left in the upper panel). Pulmonary infections are the result of initial infection through inhalation of infectious propagules (Right in the upper panel). Another manifestation is cryptococcomas (Lower panel), which is formed by an inflammatory response in brain, lungs, skin, and other organs, thus it is more common in immunocompetent hosts. It may subsequently appear in a complex granuloma, including various macrophages.* CNS* Central nervous system

The most common clinical manifestations of cryptococcal infections affect the CNS. Such infections are associated with meningitis or meningoencephalitis, which have high mortality rates (Fig. 1). Another typical manifestation in brain is the formation of cryptococcomas, which are mass lesions caused by infection of the focal tissue. The formation of cryptococcomas depends on an inflammatory response; thus, this manifestation is more common in immunocompetent individuals (Fig. 1). Cryptococcal pneumonia is usually seen when the initial infection occurs through inhalation of infectious propagules, and the involvement of other organs can ensue following the development of cryptococcemia (Fig. 1). In addition to cryptococcal pneumonia and meningitis, *C. neoformans* can also cause cutaneous cryptococcosis resulting from a primary infection of open skin wounds or a secondary infection from cryptococcal dissemination (Fig. 1).

**Fig. 2:**
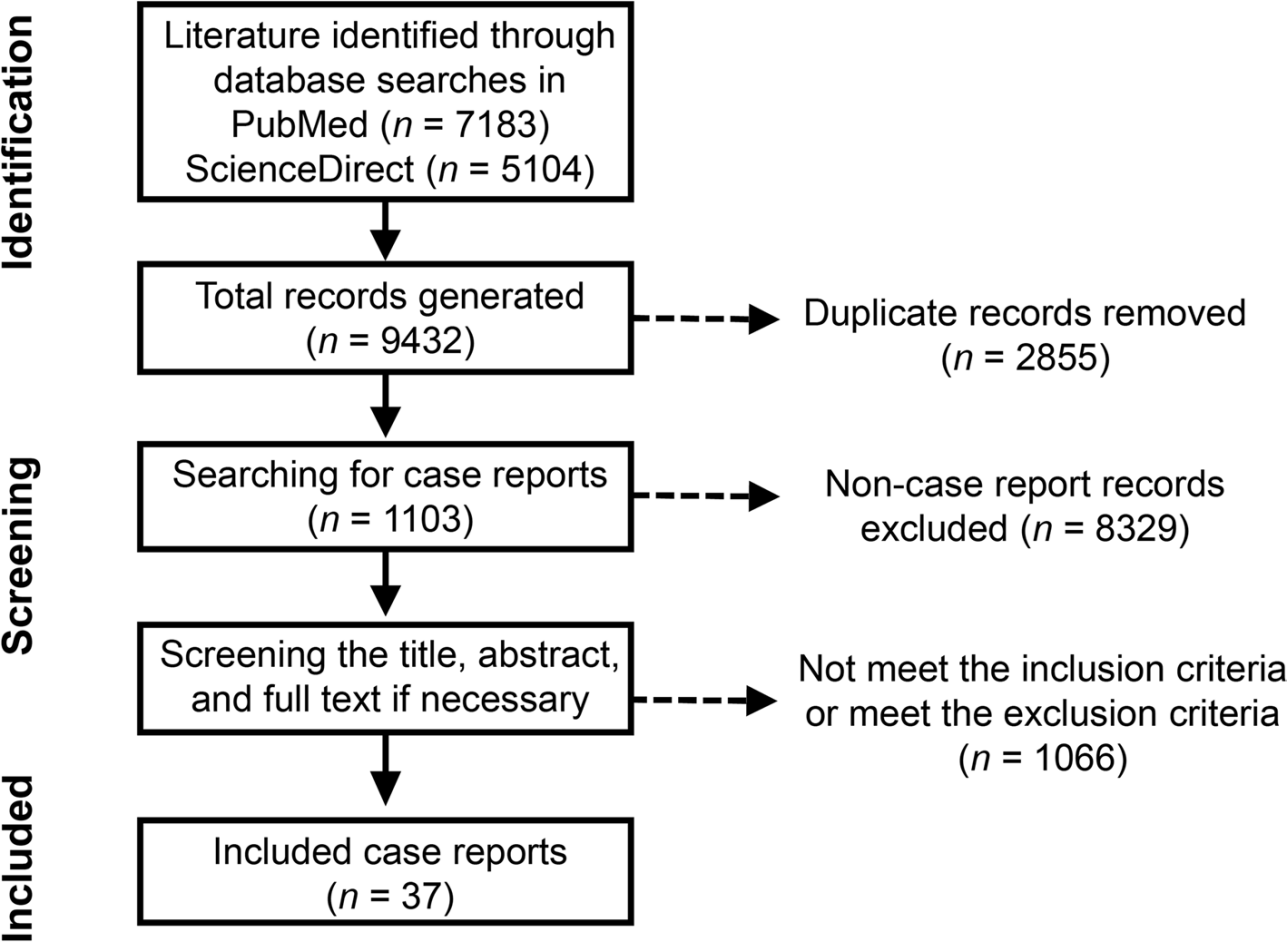
Flow diagram of the scoping review process for case reports of cryptococcosis

To obtain a systematic understanding of the clinical manifestations and features of cryptococcal infections, we reviewed case reports of cryptococcal infections during the past two decades. Of the 9432 records obtained from keyword searching during the study period, 1103 were identified as case reports. After removing excluded studies and studies that did not provide disease-specific treatment details, patient characteristics, or patient outcomes, 296 were screened for eligibility and full-text review, and 37 studies were eventually retained (Fig. 2). These 37 studies represented 38 individual patients (Table 3).

**Table 3:** Summary of 38 cryptococcosis cases since 2000

Case	Location	Age	Sex	Medical or surgical history	Clinical manifestations; duration	Treatment course	Outcome
[23]	UK	49	M	HIV infection	Fever, ulcerated lesion over left loin, dry mucous membranes, headache, vomiting; 14 days	Intravenous AmB (1 mg/kg per day) and flucytosine (100 mg/kg per day) for 2 weeks; fluconazole (400 mg/day) for 10 weeks	No neurological complications and no further episodes of opportunistic infections at 12-month follow-up
[24]	USA	25	M	HIV-infection (CD4 count less than 20 cells/µl), intermittent asthma, migraines	Altered mental state, weight loss, neck pain, cough, fever, nausea, vomiting, photophobia; 7 days	AmB and flucytosine; placement of a lumbar drain	Clinical improvement and was discharged on oral fluconazole
[25]	Australia	59	M	Autosomal dominant polycystic kidney disease, renal transplant, direct contact with cockatiel bird	Pruritic and painless rash; 1 month	LAmB and flucytosine 6 weeks; then oral fluconazole consolidation	Improvement in lower leg cellulitis, renal allograft function is stable
[26]	China	63	M	Uremia, kidney transplant	Headache, dizziness, vomiting, dyspnea, fever; 2 days	ABLC, flucytosine and voriconazole for the first 11 days; ABLC and flucytosine for 8 weeks; fluconazole for maintenance	Asymptomatic at 3-week follow-up
[27]	Iran	39	F	Relapsing remitting multiple sclerosis, two times	Headache, fever, weakness, and progressive loss of consciousness, neck stiffness with Kernig and Brudzinski signs, bilateral pupil mydriasis and papilledema	LAmB 300 mg/day and fluconazole 800 mg/day for 2 weeks; LAmB 300 mg/day and fluconazole 400 mg/day for 4 weeks; fluconazole 200 mg/day	Discharge without any major complaints after 6-week hospitalization
[28]	Spain	42	M	Diabetes, worked in public works water and sewer maintenance	5 kg weight loss and 24 h fever, 6-month history of mechanical lumbar pain	Single dose of LAmB (10 mg/kg, 870 mg) infused over 3 h, oral fluconazole (800 mg/day); fluconazole (800 mg/day for 14 days, then 400 mg/day for 1 year	No symptoms at 12-month follow-up
[29]	Cameroon	41	F	HIV-infection, gastric Kaposi’s sarcoma	Subacute occipital headaches associated with photophobia, blurred vision, phonophobia, projectile vomiting; 4 weeks	Fluconazole 1200 mg/day and flucytosine 1500 mg × 4/day	Died on day 6 of this treatment
[30]	USA	69	M	Renal transplantation recipient, diabetes, coronary artery disease, hypertension, and hyperlipidemia	Dizziness, generalized weakness, hypotension; 28 days	AmB and flucytosine for 4 days LAmB and fluconazole for 2 weeks; then oral fluconazole 400 mg/day for 8 weeks	Admitted for delirium 7 weeks after discharge and died at a 3-week hospital course due to sepsis
[22]	Vietnam	39	M	Myasthenia gravis, thymus gland removal, diabetes, HCV hepatitis, TB pneumonia, had been breeding birds	Recurrent and progressive skin lesions, mild fever, painful and persistent skin lesions with painful and swollen left knee, headache	AmB 1 mg/kg per day and fluconazole 800 mg/day for 4 weeks; fluconazole (1000 mg) for 12 months	Ulcer regression over 11 days with no exudates; discharged on day 32
[31]	Côte d’Ivoire	41	M	HIV infection	Confused, incoherent, vomiting, headache	Patient received high oral dose of fluconazole (1200 mg/day)	Died 17 days after treatment initiation
[32]	Japan	51	M	Renal transplantation recipient	Pain, high fever, erythema, ecchymosis, vesicle formation, and erosion	Lipid AmB and flucytosine	Suffered from immune reconstruction inflammatory syndrome and died
[33]	Sri Lanka	21	M	Renal transplantation recipient	Headache, low grade fever, alert, neck stiffness, bilateral symmetrical near complete ophthalmoplegia	Intravenous AmB 1 mg/kg per day for 6 weeks; then oral fluconazole 400 mg for 10 weeks	Regained full range of eye movements after 6 weeks
[34]	Saudi Arabia	67	M	Liver transplantation, cirrhosis developed severe hyponatremia, diabetes	Dizziness, confusion, ataxia, abnormal muscle movements, and leg pain, fever, drowsy, unable to follow commands	AmB for 6 weeks; then fluconazole for 1 year	Serum sodium level returned to normal baseline 3 weeks after starting AmB treatment
[35]	Japan	72	M	Ischemic heart disease 4 years prior, ICD implantation, diabetes, chronic hepatitis B	Non-productive cough and respiratory discomfort	LAmB 250 mg/day, oral flucytosine 6000 mg/day, and daptomycin 350 mg/day for 25 days	Died 54 days after initial hospital admission, an autopsy was declined by his family
[36]	China	34	M	Nephrotic syndrome	Massive shallow ulcers of both lower extremities, fever of 39 °C, severe pain	Intravenous fluconazole (400 mg/day) for 8 days; intravenous antibiotic therapy	Died 8 days after initial hospital admission
[37]	Germany	81	F	Rheumatoid arthritis	Skin ulceration, swelling, erythema and severe pain	Intravenous fluconazole (400 mg/day) for 10 days; then fluconazole (400 mg/day) for 4 weeks	The skin defect was successfully closed with a mesh graft
[38]	USA	51	M	Cardiac transplantation with mild rejection	Fever, back pain, severe left lower extremity pain	Oral fluconazole and intravenous AmB; Due to fluconazole resistance, switched to oral voriconazole and intravenous AmB	There were no fungal organisms present after treatment
[39]	China	68	M	No significant past medical history; no noted exposure to bird droppings	Progressive multiple abscesses, fever, lower extremity weakness, urinary retention; 7 months	LAmB, itraconazole, flucytosine, and fluconazole for 4 months	Afebrile, with no new-onset abscess, could walk slowly at 18-month follow-up
[40]	Thailand	70	M	With a 5-year history of primary myelofibrosis, hypertension, asthma, and osteoporosis presented	Erythematous swollen left leg for 7 days; fever, dyspnea on exertion, and orthopnea for 6 days; multiple ulcers on right foot for 10 days	AmB 50 mg/day and oral fluconazole 800 mg/day; switched to LAmB 180 mg/day and fluconazole 800 mg/day for 28 days; then oral fluconazole 400 mg/day	Developed septic shock and died 48 days after hospital admission
[41]	Japan	56	M	Hepatitis type B virus infection, hepatic failure, hypertension	Headache, vomiting, gait disturbance; 1 month	LAmB and fluconazole	Developed renal failure and ultimately died 25 days after admission
[42]	China	42	M	Hepatitis type B virus infection	Aphasia and left hemiparesis; 3 days	Intravenous fluconazole 400 mg/day; switched to intravenous AmB (0.6 mg/kg per day) and voriconazole (400 mg/kg per day) and oral flucytosine (100 mg/kg per day, 4 equal parts) for 2 weeks; then oral voriconazole for 11 months	Neurological recovery was complete and no recurrence was recorded at 6-month follow-up
[43]	Canada	56	F	Diabetes, seizure disorder with remote left temporal lobectomy	Fever up to 38.4 °C	Posaconazole 300 mg/day for 12 months	Antigen titers were negative at 1-year follow-up
[44]	Argentina	59	F	Smoking, arterial hypertension, breast cancer, nephroangiosclerosis, kidney graft	Fever, headache episodes, weight loss	Intravenous AmB and oral fluconazole	Infection resolved after antifungal treatment
[45]	Norway	54	M	Hypertension, migraine	Influenza-like symptoms, palpitations, activity-related presyncope	Intravenous fluconazole (800 mg); switched to AmB (200 mg/day) and flucytosine (1500 mg/ day) twice daily for 1 month; then flucytosine (1500 mg × 2) and fluconazole (400–800 mg/day) for 1 month; then fluconazole (400 mg/day) for 11 months	There was no evidence of infection recurrence during 2 years of follow-up
[46]	Brazil	45	M	Pulmonary tuberculosis, HIV infection	Difficulty performing basic daily life activities, anorexia, fever, profuse sweating, vomiting episodes preceded by nausea	AmB (1 mg/kg per day) and intravenous fluconazole (800 mg/day) for 7 days; then fluconazole (1200 mg/day) for 7 days; fluconazole (800 mg/day) for 8 weeks	CSF cultures for fungi were negative at 1 and 2 weeks following treatment initiation
[47]	Netherlands	60	M	HIV-negative patient with low MBL and low naïve CD4 count, chronic relapsing meningoencephali-tis with relatively mild symptoms for approximately 2 years	Relapsing diffuse headaches lasting several days; 3.5 months	LAmB (3 mg/kg per day) and flucytosine (100 mg/kg per day) for 2 weeks; fluconazole (400–800 mg/day) for 8 weeks; then fluconazole maintenance therapy for 12 weeks	Mild headaches
[48]	USA	75	M	Renal transplantation, diabetes	General weakness, altered mental status, hypoxemia, mild hyponatremia, hypochloremia	AmB and flucytosine	After an initial improvement, the patient became suddenly hypotensive, and died soon after
[49]	China	40	M	Unremarkable	Apathetic, uncommunicative and slow to move, unresponsive and bed ridden, consciousness level continued to decline during admission	Intravenous AmB 25 mg/day was given initially and then gradually increased to 50 mg/day, with oral flucytosine 6 g/day	Coma began 1 week after treatment onset; treatment was stopped according to the circumstances, and the patient died
[50]	Germany	49	F	Relapse remitting multiple sclerosis, owned a birdhouse	Cephalgia, fever, confusion, coughing and generalized weakness	Intravenous LAmB and oral fluconazole, replaced by flucytosine	Remained clinically stable
[51]	China	72	M	Chronic hepatitis B	Dry cough; more than 6 months	Fluconazole 400 mg/day for 6 months	Two pulmonary cryptococcal nodules disappeared
[52]	–	41	M	HIV-negative; alcoholic liver cirrhosis	Fever, seizure	Flucytosine	Multi-system organ failure leading to death
[53]	China	50	M	HIV infection	Multiple skin lesions, intermittent headache	AmB and fluconazole	Died 3 days after adding fluconazole
[53]	China	64	F	HIV infection, diabetes	Mild fever, productive cough, dyspnea on exertion, swelling in both lower limbs	Oral fluconazole 100 mg for 3 days; then AmB (1 mg/kg per day) for 3 days followed by fluconazole 400 mg/day	Became afebrile after 72 h of treatment with considerable improvement of other comorbidities; discharged with continuing oral antifungal therapy
[54]	China	77	M	Advanced stage non-small cell lung cancer, smoking, severe underlying chronic obstructive pulmonary disease	Non-anginal anterior chest pain, progressive dyspnea	Fluconazole 400 mg/day	Progressive clinical improvements at 10 month follow-up, the patient passed away from malignancy
[55]	China	48	F	With a 2-month history of elevated serum CA19-9 levels (50.05 IU/ml)	No symptoms	Fluconazole 400 mg/day for 3 months; fluconazole 200 mg/day for 3 months	After 6 months of treatment, serum CA19-9 levels regressed to the normal range

*AmB* Amphotericin B;* LAmB* Liposome bilayer-coated amphotericin B;* ABLC* Amphotericin B lipid complex;* HCV* Hepatitis C virus; TB Tuberculosis;* ICD* implantable cardioverter-defibrillator;* CSF* Cerebrospinal fluid;* CA19-9* Carbohydrate antigen 19-9

The age of the patients ranged from 21 to 81 years (median 51 years), and 71% (*n* = 27) were male. Of the 38 patients, 95% (*n* = 36) reported a medical or surgical history, 21% (*n* = 8) were infected with HIV, 18% (*n* = 7) had a history of organ transplantations (6 renal transplantations, 1 liver transplantation, and 1 orthotopic heart transplantation), 21% (*n* = 8) had diabetes mellitus, and 18% (*n* = 7) had liver hepatopathies (5 hepatitis B infection and 2 liver cirrhosis). It is worth mentioning that one patient had been breeding birds [22]. In the identified cases, patients with cryptococcal infections most commonly presented with fever (50%, *n* = 19), headache (29%, *n* = 11), and vomiting (21%, *n* = 8). Less common manifestations included altered mental status and/or confusion (13%, *n* = 5), cough (13%, *n* = 5), and drowsiness or fatigue (8%, *n* = 3). On physical examination, 11% (*n* = 4) were noted to have body weight loss, and 11% (*n* = 4) were observed to have weakness in the extremities. Time from symptom onset to hospital presentation ranged from 2 to 210 days. Even though all 38 patients received systemic antifungal treatment, the mortality was as high as 34% (*n* = 13); over half (7/13) of the deaths were associated with brain infections. Patients’ ages at death ranged from 30 to 75 years (median 45.5 years). Time from hospital presentation or symptom onset to death ranged from 2 to 420 days (median 25.5 days).

Our scoping review in this section included cryptococcal infections associated with the brain, lungs, skin, and other organs in both HIV-positive and HIV-negative cases. This diversity of infection conditions resulted in deviations in clinical manifestations and statistics as compared with HIV-associated CM, which tends to attract the attention of healthcare professionals. To provide a more concentrated analysis, we review the recent research progress focusing mainly on CM in the following sections; we apologize to those whose work could not be properly discussed and cited.

### Diagnosis and management of CM

The key reasons for the high mortality of CM include: (1) delays in diagnosis, largely as a result of limited access to lumbar puncture (LP) and rapid diagnostic assays; (2) the limited availability and high cost of currently recommended antifungal agents and intensive care; and (3) the limited ability to monitor and manage treatment-limiting toxicity and the increased intracranial pressure that is frequently associated with CM. Therefore, improving diagnostic abilities and developing more effective treatments would reduce the mortality associated with CM. In this section, we summarize current updates in the diagnosis and antifungal treatment of CM.

#### Diagnosis

**Fig. 3:**
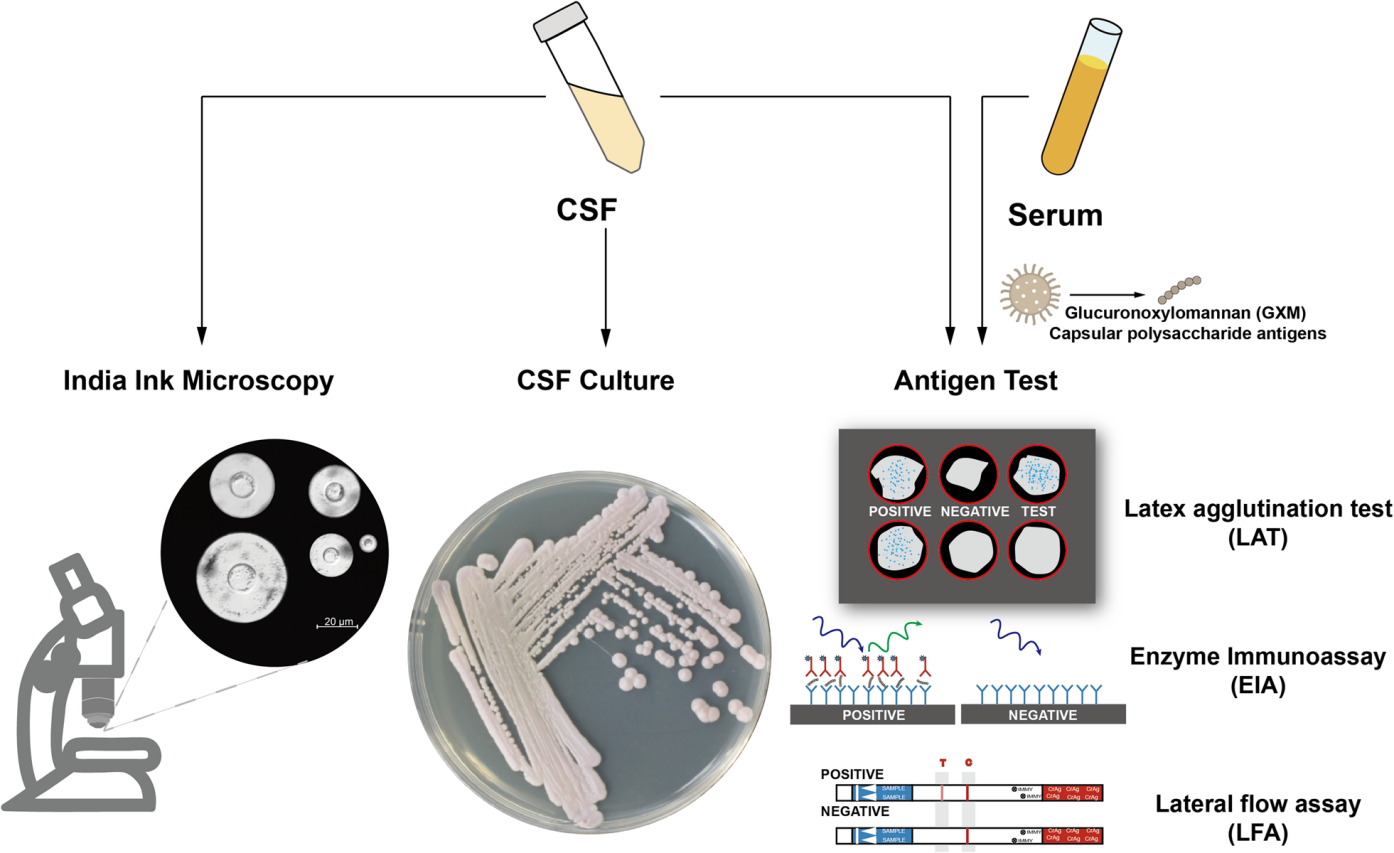
Diagnosis of cryptococcal infections. India ink microscopy, cerebrospinal fluid (CSF) culture, and detection of cryptococcal antigen in serum or CSF are three protocols for diagnosis of cryptococcal infections. India ink microscopy remains the primary diagnostic tool for identifying Cryptococcus in CSF. CSF fungal culture is the gold standard for diagnosis of cryptococcal meningitis. The detection of cryptococcal antigens, the capsular polysaccharide glucuronoxylomannan (GXM), is a very sensitive, specific, and effective test to detect cryptococcal infections. The antigen test was mainly performed through the Latex agglutination test (LAT), Enzyme Immunoassay (EIA) and Lateral flow assay (LFA).

Several protocols are now available for the diagnosis of CM in HIV-infected patients, including India ink microscopy, cerebrospinal fluid (CSF) culture, and detection of cryptococcal antigen in serum or CSF (Fig. 3) [56, 57]. The use of India ink microscopy remains the primary diagnostic tool for identifying *Cryptococcus* in the CSF. Although India ink microscopy is readily available, it is associated with a low sensitivity of approximately 70–90% [58], particularly in patients with low fungal burdens. Thus, the use of India ink microscopy as the sole diagnostic tool could result in misdiagnosis, particularly soon after symptom onset or in patients undergoing antiretroviral therapies.

CSF fungal culture is the gold standard for the diagnosis of CM, and a positive culture usually implies active cryptococcal disease. It also produces false negative results, similar to India ink microscopy, when the fungal burden is low. To partially overcome this drawback, the CSF volume applied for quantitative fungal culture has been modified from 10 µl to 100 µl in an updated culture protocol [59]. This change led to an improvement of the diagnostic sensitivity of CSF fungal culture from 82.4% to 94.2%. Additional intrinsic drawbacks of the quantitative CSF fungal culture are the general slow-growing nature of the fungus as well as a specific physiological status of *Cryptococcus* called the viable-but-nonculturable (VBNC) state [60]. Because of these factors, obtaining a quantitative result may require several weeks of culture, and a percentage of cells may not be successfully cultured. In addition, fungal culture requires proper laboratory settings and trained technicians. Nevertheless, quantitative CSF fungal culture remains central for the definitive diagnosis of CM.

LP followed by India ink microscopy or CSF fungal culture is often deferred until the disease is advanced. The detection of cryptococcal antigens, such as the capsular polysaccharide glucuronoxylomannan (GXM) in serum or CSF, has become an essential diagnostic approach and is used for presumptive diagnosis. It is a very sensitive, specific, and effective test that can detect the infection early, ahead of symptom onset and before the disease can develop into life-threatening CM.

Antigen tests are mainly performed in the form of latex agglutination tests (LAT) or enzyme immunoassays (EIA), which are sensitive, specific, and readily available from commercial sources. However, both tests require appropriate laboratory infrastructure and trained technicians, and immunoassay tests tend to be too expensive to allow routine use in resource-limited regions. A major advance, the cryptococcal antigen lateral flow assay (LFA), has revolutionized the diagnosis of CM, particularly in resource-limited settings [61]. LFA is stable at room temperature, requires no specimen preparation, provides results in minutes, and is 100-fold more sensitive to capsular polysaccharides than that of LAT [62, 63]. More significantly, it can be used to detect the cryptococcal antigen in versatile sample types, such as serum, CSF, plasma, and urine, enabling early diagnosis of CM even in facilities where LP or blood sampling is not feasible. Furthermore, semi-quantitative LFA titers have been developed for gross approximation of the fungal burden [64–66]. Further studies are needed to investigate the use of semi-quantitative LFA titers for screening potential infections and monitoring treatment responses.

Diagnosis of CM should be relatively easy in HIV-infected patients, given the high fungal burden. The WHO guidelines from 2018 recommended that HIV-infected adults and adolescents who have a CD4 cell count less than 100 cells/µl should be screened for cryptococcal antigen, and the CD4 cell count threshold was expanded to less than 200 cells/µl in the 2022 WHO guidelines. The preferred diagnostic approach recommended in the WHO guidelines of 2022 is prompt LP with measurement of CSF opening pressure and rapid cryptococcal antigen assay. LP with CSF India ink microscopy is the alternative approach, only if access to a cryptococcal antigen assay is not available or rapid results cannot be obtained. Under settings without immediate access to LP or when these diagnostic approaches are clinically contraindicated, rapid serum, plasma, or whole-blood cryptococcal antigen assays are the preferred diagnostic approaches.

CM recognition in immunocompetent and non-HIV-infected cases can be challenging due to the low early fungal burden, which makes fungal culture and LAT techniques less sensitive. In addition, the indolent presentation and subacute nature of symptoms often lead to late diagnoses and consequent disease severity. The improved sensitivity makes the LFA test the preferred diagnostic approach in this context. A low threshold for suspected CM is helpful in the management of the disease, and the use of accessible diagnostic assays to perform early diagnoses is required to achieve lower rates of morbidity and mortality.

#### Antifungal treatments

Antifungal treatments of IFDs in current clinical practice are limited to only four classes of systemic antifungal agents (azoles, polyenes, pyrimidines, and echinocandins). The limitation in antifungal treatment options for invasive cryptococcal infection is particularly significant, given that *Cryptococcus* species have intrinsic resistance to echinocandins, and some clinical isolates have been found to acquire resistance to azoles [67, 68]. Therefore, the polyene amphotericin B (AmB) has been prescribed as the primary antifungal drug for the management of cryptococcal infections, despite its toxicity and the very high cost of less toxic formulations [69].

The management of CM is divided into three phases: (1) induction, (2) consolidation, and (3) maintenance of antifungal treatment regimens. The WHO guidelines of 2018 [70] for the treatment of cryptococcal disease in patients infected with HIV recommended a 1-week induction regimen with AmB deoxycholate (1.0 mg/kg per day) and flucytosine (100 mg/kg per day, divided into four daily doses), followed by 1 week of fluconazole (1200 mg/day for adults, 12 mg/kg per day for children and adolescents up to a maximum dose of 800 mg/day); an 8-week consolidation regimen with fluconazole (800 mg/day for adults, 6–12 mg/kg per day for children and adolescents up to a maximum dose of 800 mg/day) following the induction phase; and a maintenance regimen with fluconazole (200 mg/day for adults, 6 mg/kg per day for adolescents and children) until immune reconstitution [70].

Various formulations of AmB are commercially available, including liposomal, deoxycholate, and lipid complex formulations. Of note, these formulations are not interchangeable [71], and only AmB deoxycholate and liposome bilayer-coated AmB (LAmB) have been recommended for managing CM. The WHO guidelines of 2018 raised the possibility that LAmB could be preferrable as a formulation over AmB deoxycholate, considering its equivalent efficacy and improved safety [72]. In March 2022, a phase 3 randomized, controlled, noninferiority trial conducted in five African countries concluded that single-dose LAmB combined with flucytosine and fluconazole was non-inferior to the treatment recommended in the 2018 WHO guidelines for HIV-associated CM and was associated with fewer adverse events [73]. Also considering the extrapolation of evidence supporting the use of a single high dose (10 mg/kg) of LAmB to children [74, 75], the WHO Guideline Development Group updated the recommendations in the 2022 WHO guidelines. These newer guidelines include a single high-dose LAmB-based regimen with 14 days of flucytosine and fluconazole as the preferred induction therapy for managing CM, while previously recommended alternative regimens remain valid [76]. Unfortunately, this updated regimen exacerbates the difficulty of accessing a referred treatment that is already posed by flucytosine, which is expensive and not always available in resource-limited countries. Future efforts should be directed towards improving the accessibility and affordability of flucytosine in low- and middle-income countries with high infection loads.

### Antifungal resistance and tolerance in *C. neoformans*

**Fig. 4:**
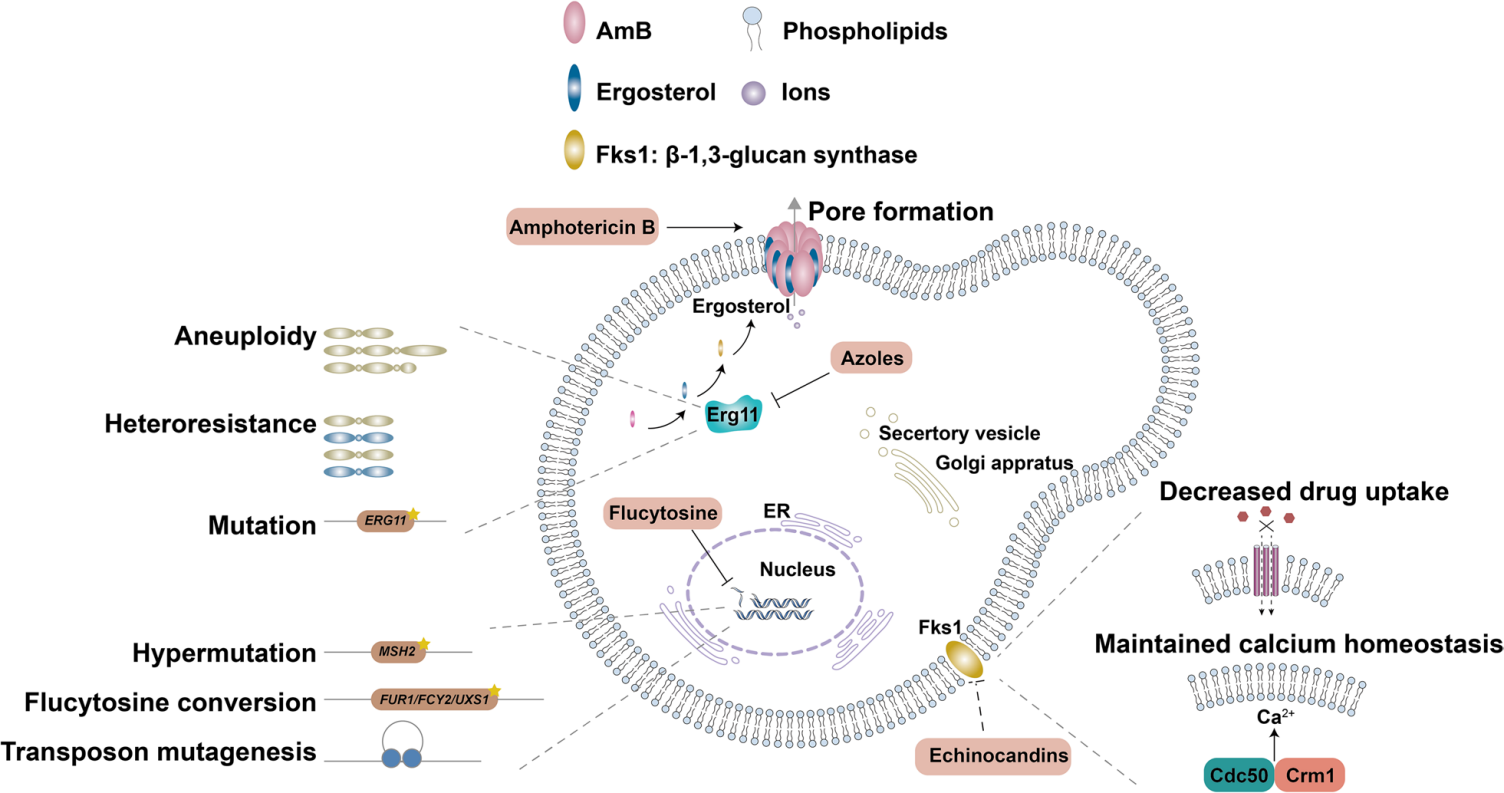
Antifungals, targets, and antifungal resistance mechanism. Three main classes of drugs are used in the treatment of cryptococcal infections: polyenes (AmB), azoles (fluconazole), flucytosine. *C. neoformans* is intrinsic resistant to echinocandins (shown in dotted lines). AmB binds to ergosterol in the cell membrane, which forming pores and exerting fungicidal activity. Fluconazole targets the ergosterol biosynthetic enzyme *Erg11*. Flucytosine blocks DNA synthesis. *C. neoformans* cells develop resistance to different drugs through different mechanisms. The exact mechanism leading to AmB resistance is not yet clear. Fluconazole resistance has been reported to be associated with aneuploidy, heteroresistance and mutations in the *ERG11* gene. Flucytosine resistance may be associated with mutations in genes related to flucytosine conversion, hypermutation, and transposon mutagenesis. The echinocandins resistance is attributed to the interaction of Cdc50 and Crm1, and preventing drug uptake could also arise echinocandins resistance

*Cryptococcus neoformans* is susceptible to polyenes, flucytosine, and azoles, which are clinically used together in the three-phase therapy of CM. The polyene AmB was the first antifungal drug developed to treat systematic fungal infection, and it is fungicidal rather than fungistatic [77, 78]. Mechanistically, AmB binds to ergosterol-containing membranes, which are the major membranes found in fungal cells, yielding pores in membranes and exerting antifungal activity (Fig. 4) [79]. Acquisition of resistance to AmB by *C. neoformans* has rarely been reported, but the detection of AmB resistance can be technically challenging, and the true rate of AmB resistance is not known [78, 80, 81]. Nevertheless, cryptococcal isolates with altered AmB sensitivity have been reported [82–85]; however, the exact mechanisms leading to these changes remain unknown. Cryptococcal AmB resistance has been shown to be caused by alterations in ergosterol biosynthesis through mutations in sterol Δ8-7 isomerase [82–84, 86]. On the other hand, AmB resistant isolates without altered ergosterol biosynthesis have also been reported [87, 88], indicating the existence of alternative mechanisms that confer AmB resistance in *C. neoformans*.

Despite the rarity of AmB-resistant strains, outcomes of AmB therapy appear to be unsatisfactory, and relapses of cryptococcal infections are common; therefore, further investigation into the mechanisms of interaction between fungi and antifungal agents is warranted. In bacteria, both bactericidal resistance and tolerance can affect the outcome of antibiotic therapy. Unlike bacteria with genetically heritable resistance that can replicate in the presence of a drug at concentrations above the minimum inhibitory concentration (MIC), bacteria that are considered “tolerant” are genetically susceptible and can withstand the killing effect of high doses of bactericidal antibiotics. Time-kill curve-based assays are used to evaluate bacterial tolerance to microbicidal antibiotics. In the context of *Cryptococcus*, Rodero et al. found a correlation between the time-kill curve of AmB and the clinical outcomes of 16 patients with cryptococcal meningitis [89]. A correlation between the time-kill curve and the clinical outcome was also reported by Córdoba et al., whose study encompassed a larger number of patients and isolates (74 clinical strains isolated from 60 patients) [90]. These findings suggest that cryptococcal tolerance to fungicidal AmB may have an impact on the therapeutic outcome of cryptococcal meningitis.

Flucytosine was first chemically synthesized in 1957 as a potent antibacterial and antitumoral compound [91]. As a prodrug, it enters cells via cytosine permease Fcy2, and its function depends on its conversion to 5-fluorouracil by cytosine deaminase Fcy1 and further processing by the uracil phosphoribosyltransferase Fur1. The converted metabolites then inhibit thymidylate synthase activity and consequently inhibits DNA and RNA synthesis [92, 93]. The emergence of resistance to flucytosine prevents its use as a monotherapy drug for fungal infection treatment [92, 94–97], and it is used in combination with AmB as a first-line induction treatment for cryptococcal infections [76, 98, 99].

Although flucytosine resistance has been well-studied in *Candida* spp. [100–102], little work has been done to investigate resistance in *C. neoformans*. It has been reported that nonsense mutations within the DNA mismatch repair protein coding gene *MSH2* confer flucytosine resistance in clinical isolates of *C. neoformans* [103]. This study clearly showed that hypermutator phenotypes are associated with the acquisition of resistance to antifungals, including flucytosine, in *C. neoformans*. Billmyre et al. demonstrated that DNA mismatch repair defects enable rapid acquisition of resistance to flucytosine in *C. deuterogattii*, the sister species of *C. neoformans*, and they further identified important mutations in known resistance genes (*FUR1* and *FCY2*) and a capsule biosynthesis-related gene *UXS1* [104]. This study provides direct evidence that support the recent appreciation that hypermutation may be a common mechanism that accelerates the acquisition of antifungal resistance in pathogenic fungi. In addition, transposon mutagenesis has been shown to be a contributor to the acquisition of flucytosine resistance during the environment-to-host transition in *C. neoformans* [105]. While these studies identified potential resistance-related mechanisms that influence flucytosine conversion (Fig. 4), further study is needed to clarify mechanisms leading to flucytosine resistance.

Fluconazole is the most commonly used antifungal agent for the treatment of cryptococcal infections (Fig. 4). It inhibits fungal ergosterol biosynthesis via binding to the cytochrome P450 enzyme sterol 14-demethylase (Erg11 or Cyp51), leading to disrupted cell membrane integrity [106]. Given that an 8-week consolidation regimen of fluconazole followed by a maintenance regimen of low-dose fluconazole is recommended by the 2022 WHO guidelines [76] for CM treatment, the prolonged use and changes in recommended dosages exerted selection pressure and contributed to the prevalence of fluconazole resistance in cryptococcal clinical isolates [107]. A series of thresholds of fluconazole susceptibility has been established to classify clinical isolates: an isolate that exhibits an MIC of most 8 µg/ml is considered susceptible, an isolate with an MIC of 16–32 µg/ml is considered dose-dependent susceptible, and an isolate with an MIC of at least 64 µg/ml is considered resistant [104, 107].

Heteroresistance to fluconazole and other azole antifungal agents is clinically ubiquitous (Fig. 4), and it contributes to the relapse of cryptococcosis during fluconazole maintenance therapy [103, 108]. *C. neoformans* is innately heteroresistant to fluconazole [109], which primarily occurs by transient duplications of chromosomes [110, 111]. Chromosome 1, which harbors the genes *ERG11* and *AFR1* (encoding an ABC transporter), is the first chromosome to be duplicated at fluconazole levels higher than the MIC [110], and further increases in drug levels result in the disomy of chromosome 4, which contains *SEY1* (encoding a GTPase), *GLO3* and *GCS2* (encoding the ADP-ribosylation factor GTPase activating proteins) [112]. The duplicated chromosomes or aneuploidy can be readily lost during maintenance in drug-free conditions [110]. Fluconazole resistance in *C. neoformans* has also been associated with mutations in the *ERG11* gene [113, 114]. It is worth to mention that new-generation triazole antifungals with higher activities against resistant and emerging fungal pathogens have been developed either from fluconazole or itraconazole, such as voriconazole, posaconazole, and isavuconazole [115]. The new-generation triazoles have been tested for the treatment of invasive aspergillosis and candidiasis [116], indicating clinical implications for CM treatment.

*C. neoformans* is intrinsically resistant to echinocandins (Fig. 4), which is paradoxical, as the inhibitory target of echinocandins (β-1,3-glucan synthase) is essential in *Cryptococcus* [117–119]. Huang et al. discovered that a mutation in *CDC50*, which encodes the β-subunit of membrane lipid flippase, can mediate echinocandin resistance via preventing drug uptake in *C. neoformans* [117]. Forward genetic screening for *cdc50*Δ suppressor mutations led to the identification of a homolog of the mechanosensitive channel protein Crm1 that is involved in Cdc50-mediated caspofungin resistance [120]. Cdc50 interacts with Crm1 to regulate calcium homeostasis and caspofungin resistance via calcium/calcineurin signaling [121].

Given the rapid emergence of antifungal resistance and the lack of treatment options, advances in technology to detect antifungal resistance and research focused on understanding antifungal resistance mechanisms will contribute to develop novel antifungal drugs and therapeutic strategies.

## Conclusion

The rising threat of *C. neoformans* is compounded by the accumulating evidence for its capability to infect immunocompetent individuals and the emergence of antifungal-resistant variants. More global surveillance data on antifungal susceptibility combined with molecular typing of *C. neoformans* would facilitate the correlation of antifungal resistance or tolerance with different genotypes, enabling the use of genotyping strategies to permit data-driven evaluation of risk and promoting the development of corresponding treatment strategies. The construction and integration of robust fungal disease surveillance systems would permit the development of a detailed understanding of global and local epidemiology of *C. neoformans* and other fungal pathogens, which is especially important for hyper-virulent or drug-tolerant variants. In addition, developing systematic approaches to comprehensively explore the mechanisms of fungal pathogenicity and antifungal resistance and tolerance promises to lead to the identification of new targets for antifungal drugs and the development and optimization of new and existing diagnostic and therapeutic approaches, thus providing important safeguards to reduce the morbidity and mortality of fungal infections.

## Data Availability

All data generated or analyzed during this study are included in this published article.

## Abbreviations

WHO: World Health Organization
FPPL: Fungal Priority Pathogens List
PRISMA-ScR: Preferred reporting items for systematic reviews and meta-analyses extension for scoping reviews
IFD: Invasive fungal disease
HIV: Human immunodeficiency virus
HAART: Highly active antiretroviral therapy
AIDS: Acquired immunodeficiency syndrome
CM: Cryptococcal meningitis
CNS: Central nervous system
CSF: Cerebrospinal fluid
CrAg: Cryptococcal antigen
LP: Lumbar puncture
GXM: Glucuronoxylomannan
LAT: Latex agglutination test
EIA: Enzyme immunoassay
LFA: Lateral flow assay
AmB: Amphotericin B
LAmB: Liposome bilayer-coated amphotericin B
ABLC: Amphotericin B lipid complex
MIC: Minimum inhibitory concentration
MBL: Monoclonal B-cell lymphocytosis
CD: Cluster of differentiation
HCV: Hepatitis C virus
TB: Tuberculosis
ICD: Implantable cardioverter-defibrillator
MBL: Monoclonal B-cell lymphocytosis
CA19-9: Carbohydrate antigen 19-9
